# Galactose-Induced Cataracts in Rats: A Machine Learning Analysis

**DOI:** 10.7150/ijms.103892

**Published:** 2025-02-10

**Authors:** Ahmed Jasim Mahmood Al-Mashhadani, Qi Gong, Franko Shehaj, Lianhong Zhou

**Affiliations:** 1 Department of Ophthalmology, Renmin Hospital of Wuhan University, Wuhan 430060, Hubei Province, P.R. China.; 2 Department of Orthopedics, Renmin Hospital of Wuhan University, Wuhan 430060, Hubei Province, P.R. China.

**Keywords:** eye, cataract, rat, decision tree, PCR

## Abstract

**Background:** Rat models are widely used to study cataracts due to their cost-effectiveness and prominent physiological and genetic similarities to humans The objective of this study was to identify genes involved in cataractogenesis due to galactose exposure in rats.

**Methods:** We analyzed four datasets from the Gene Expression Omnibus, including both *ex vivo* and *in vivo* models of cataracts in different rat strains. Feature selection tools were used to identify genes potentially relevant in cataract-related gene expression. A decision tree algorithm was implemented, and its predictions were interpreted using SHAP and LIME. To validate gene expression levels, PCR was conducted on six rat lenses cultured in M199 medium and galactose to induce cataract and six lenses cultured in M199 alone.

**Results:** Using feature selection tools, four key genes—PLAGL2, CMTM7, PCYT1B, and NR1D2—were identified. Only PCYT1B was significantly differentially expressed between the cataract and control groups across analyzed datasets. The model showed strong predictive performance, particularly in *ex vivo* datasets. SHAP and LIME analyses revealed that CMTM7 had the largest impact on model predictions. PCR results did not show significant differences in gene expression between the cataract and control groups.

**Conclusion:** The decision tree model trained on an *in vivo* dataset could predict *ex vivo* and *in vivo* cataracts despite no significant gene expression differences found between the cataract and control groups. Given a small number of samples, larger studies are needed to validate our findings.

## Introduction

Cataract is a common ocular condition characterized by the gradual clouding of the lens, which leads to impaired vision. It is a leading cause of blindness worldwide, affecting millions of people and significantly impacting the quality of life^1^, ^2^. The primary risk factors for cataract development include aging, use of medications, such as corticosteroids, smoking, alcohol consumption, and certain systemic diseases, such as diabetes mellitus^3^, ^4^. The etiology of cataracts is multifactorial, involving a combination of genetic, environmental, and lifestyle factors. Oxidative stress, protein aggregation, alterations in lens metabolism, and many other mechanisms have been implicated in the pathogenesis of cataract, leading to the formation of opacities that obstruct light transmission through the lens^5^.

Numerous animal models have been used to study cataractogenesis and evaluate potential therapeutic interventions^6^-^9^. Among these, rat models are widely used due to their cost-effectiveness and prominent physiological and genetic similarities to humans^10^, ^11^. Cataracts in rat models can be induced through various methods. The galactose-induced cataract model, both *in vivo* and *ex vivo*, is particularly notable. In the *in vivo* approach, rats are administered a high-galactose diet, which leads to the development of cataracts over time. This method mimics the gradual onset of cataracts as seen in human patients with diabetes mellitus. The *ex vivo* model involves incubating isolated rat lenses in a galactose solution, which induces cataract formation more rapidly and allows for controlled examination of lens pathology^12^. Moreover, specific rat strains, such as Lewis, Royal College of Surgeons (RCS), Sprague-Dawley (SD), Ihara Cataract Rat (ICR), and others, are invaluable in ophthalmic research^13^-^17^. Rat strains such as ICR (not to be confused with ICR mice, which were developed by the Institute of Cancer Research), which are exclusively used by Japanese researchers, make a great model for studying cataracts as they are prone to the spontaneous development of this disorder^18^.

The association between cataracts and various systemic diseases, particularly diabetes, has been known for a while^3^. Diabetes mellitus is a well-established risk factor for cataract formation, with diabetic patients showing an increased prevalence of cataracts compared to non-diabetic individuals^19^. The hyperglycemic environment accelerates lens protein glycation and oxidative stress, leading to the formation of cataracts^20^. Research utilizing rat models has been pivotal in identifying the mechanisms by which diabetes contributes to cataract development, including the role of altered glucose metabolism and inflammatory pathways^21^. In diabetic cataract models, hyperglycemia induces oxidative stress and polyol pathway activation, leading to sorbitol accumulation in the lens. This osmotic stress causes lens fiber cell swelling and rupture^22^-^24^. Additionally, advanced glycation end-products form on lens proteins, altering their structure and function, which contributes to lens opacity^20^. Inflammation is also exacerbated by upregulation of pro-inflammatory cytokines such as TNF-α and IL-1β, further damaging lens cells^25^, ^26^.

Advances in high-throughput technologies have enabled the comprehensive analysis of gene expression changes in tissues, revealing potential biomarkers and therapeutic targets^27^-^29^. Various bioinformatics approaches can be employed to analyze gene expression data, including differential expression analysis and machine learning techniques^30^, ^31^. Machine learning algorithms are increasingly being integrated into ophthalmological applications, such as cataract diagnosis^32^. In this study, we utilized the Least Absolute Shrinkage and Selection Operator (LASSO) and Random Forest (RF) algorithms to identify the most relevant genes associated with cataract. These methods are effective in reducing dimensionality and selecting features that significantly contribute to the model's predictive performance^33^. By applying these techniques, we aimed to pinpoint genes that are associated with cataract development and assess their relevance through decision tree (DT) algorithm training and validation. The application of machine learning algorithms can uncover complex patterns and interactions that may not be apparent through traditional statistical methods^31^, ^34^. This approach can help identify key genes involved in cataractogenesis and assess their potential utility as biomarkers or therapeutic targets.

## Methods

### Data Collection

**Figure 1:**
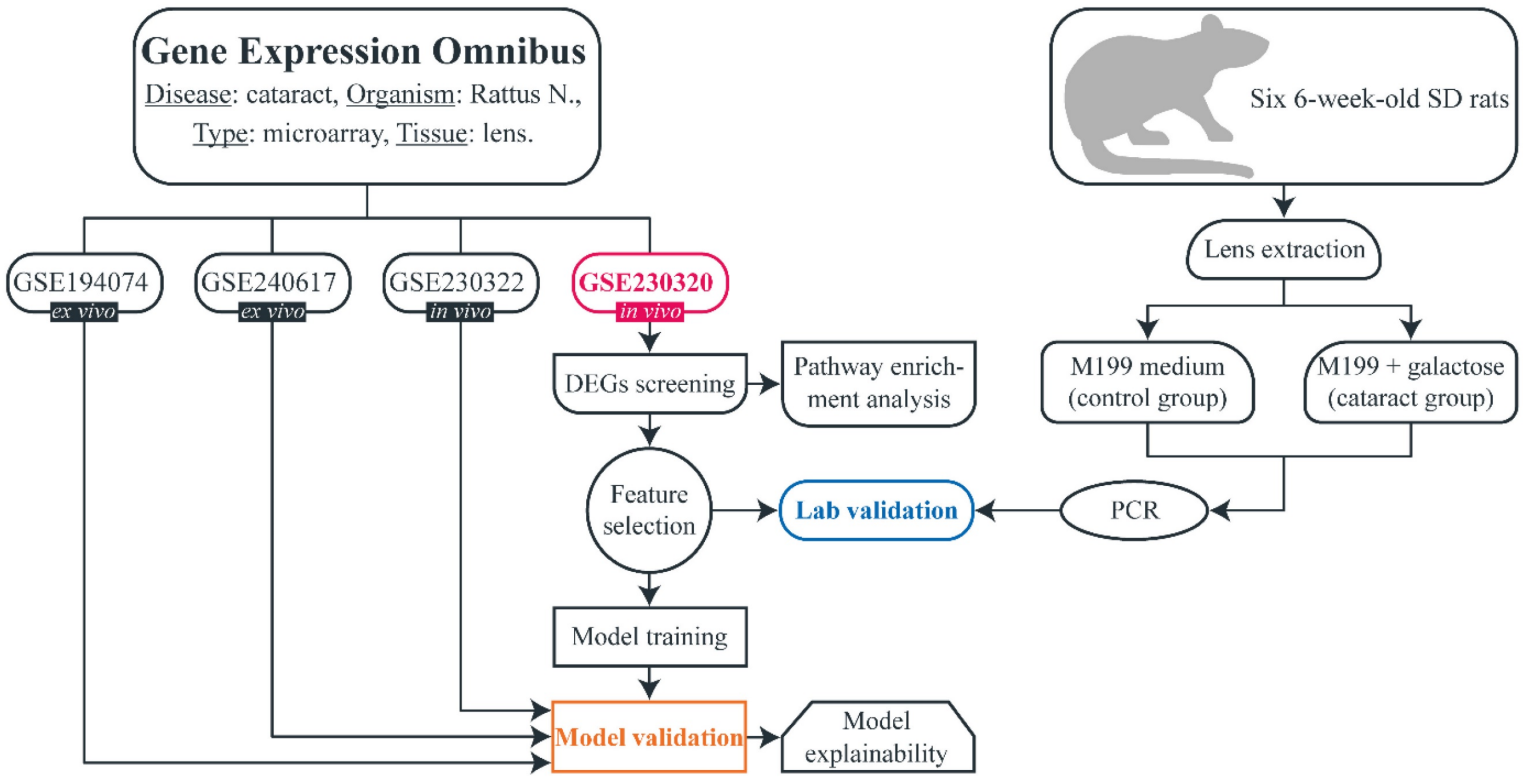
Flowchart of the study.

The flowchart of the study is shown in Fig. 1. A search (from inception until 10^th^ April) was conducted in the Gene Expression Omnibus (GEO) to download gene expression datasets. The inclusion criteria were as follows: availability of both cataract and control samples, availability of raw unprocessed data, more than two samples in the dataset, experiment type: array, organism: Rattus norvegicus, extracted material: lens tissue. Four datasets were identified: GSE194074, GSE240617, GSE230320, and GSE230322. All microarray experiments were conducted using Affymetrix Rat Gene 2.0 ST Array, platform - GPL17117). All datasets included galactose-induced cataract samples and non-cataract (control) samples. Samples that were subjected to any treatment after inducing cataract were not included in our study. GSE194074 and GSE240617 were conducted *ex vivo* and consisted of four lens samples (three cataract and one control) and five samples (two cataract and three control), respectively. GSE230320 and GSE230322 were performed *in vivo* and included seven lens samples (five cataract and two control) and 12 samples (six cataract and six control), respectively. We hypothesized that there are no major phenotypical differences between non-cataractous lenses from SD and ICR rats, and therefore, no samples were excluded based on this consideration. In addition, we wanted to determine whether the model can successfully predict cataracts regardless of age and strain. Detailed information on each dataset is provided in Table 1.

R v4.4.1 (Bioconductor v3.19, BiocManager v1.30.23) was utilized to download and normalize datasets as well as perform basic bioinformatics analysis. Datasets were downloaded via the GEOquery package (v2.72.0). Each dataset was subjected to background correction, normalization, and log2 transformation using the RMA function (oligo package, v1.68.2). Then, datasets were annotated with pd.ragene.2.0.st (annotateEset function available in the affycoretools package, v1.76.0). Rows with missing gene symbols and rows with multiple gene symbols were removed. The average value of each column was calculated for every group of rows that shared the same gene symbol.

### Bioinformatics Analysis

GSE230320 was regarded as a discovery dataset and was used for differential expression analysis and model training. limma package (v3.60.4) was employed to conduct differential expression analysis. lmFit followed by eBayes and topTable (Benjamini-Hochberg procedure adjusted) functions were utilized to identify differentially expressed genes (DEGs). DEGs with P-value < 0.05 and log fold change (FC) > 0.4 were considered upregulated and DEGs with P-value < 0.05 and log FC < -0.4 were considered downregulated. The density plot of log FC was constructed using basic graphics in R, and the volcano plot was built with the EnhancedVolcano package (v1.22.0).

Gene Ontology (GO) and Kyoto Encyclopedia of Genes and Genomes (KEGG) pathway enrichment analyses were conducted using enrichGO and enrichKEGG functions available in the clusterProfiler package (v4.12.2) and the org.Rn.eg.db package (v3.19.1). Alluvial plot was constructed with the help of ggalluvial (v0.12.5) and ggplot2 (v3.5.1) packages.

### Identification of Relevant Genes

Machine learning analysis, including feature selection, was conducted in Python (v3.12.5) with scikit-learn library. LASSO (via LassoCV) and RF (via RandomForestClassifier) were utilized to reduce the dimensionality of the dataset and improve the performance of machine learning algorithms by focusing only on the most important genes. In short, LASSO selects features by shrinking less important ones to zero, thus removing them from the final model, whereas RF selects features by building multiple decision trees and measuring how much each feature improves the accuracy of the final model. GridSearchCV was employed to identify the optimal number of features. Genes selected by LASSO and RF were intersected. The rationale for this is that intersecting genes selected by these two methods could increase the likelihood of identifying truly relevant genes. Each method has its strengths and weaknesses, so intersecting results helps filter out noise and biases specific to one method.

A Venn diagram was drawn to identify the overlapping genes. A T-test was conducted to calculate differences in the expression levels of overlapping genes between cataract and control samples in each dataset (rstatix package, v0.7.2). A P-value of <0.05 was considered statistically significant. Violin plots and heatmap were created with the ggplot2 and pheatmap (v1.0.12) packages, respectively.

### Machine Learning Analysis

DT (DecisionTreeClassifier) was trained on the GSE230320 dataset using the intersected genes identified by LASSO and RF. Hyperparameter tuning was performed using GridSearchCV. GSE194074, GSE240617, and GSE230322 were used to validate the final model. The area under the receiver operating characteristics curve (AUROC, or AUC) was calculated to evaluate the performance of DT for each validation dataset.

To enhance the interpretability of the final model, we employed three distinct methods: permutation feature importance (PFI), SHapley Additive exPlanations (SHAP), and Local Interpretable Model-agnostic Explanations (LIME). PFI was utilized to assess the contribution of each gene to the model's predictive performance. This method involves shuffling the values of each gene and measuring the impact on model accuracy^35^. SHAP values provide a unified measure of feature importance and their effects on the prediction for individual instances. By calculating Shapley values, which are derived from cooperative game theory, SHAP explains how each gene contributes to each prediction. LIME was applied to generate local explanations for individual predictions. LIME shows how the model arrives at specific predictions by highlighting the influence of each gene locally^36^.

### Sample Collection

An *ex vivo* experiment was performed in order to validate the expression of the identified genes. Six 6-week-old male SD rats (purchased from Hubei Laboratory Animal Research Center) were sacrificed by cervical dislocation. Lenses were removed using aseptic techniques and placed in M199 culture solution containing 100 U/mL penicillin and 100 µ/mL streptomycin. Then, they were incubated at 37℃ in a 5% CO_2_ incubator for 6 hours. Lens tissues were examined, and those that were not injured and remained transparent were selected for the subsequent experiments.

The 12 lenses were randomly divided into two groups: the cataract group, in which the lenses were cultured in M199 medium containing 30 mmol/galactose, and the control group, in which the lenses were cultured in M199 medium without galactose. Six lenses in each group were cultured at 37℃ with 5% CO2 for 48 hours. Lenses in both groups were carefully examined for any cataractous changes.

### Real-Time Polymerase Chain Reaction

First, 1 ml of RNA extraction buffer and three 3 mm grinding beads were added into the grinding tube, which was then placed on ice to chill. The lens was placed into the grinding tube, and the total RNA was extracted according to the manufacturer's instructions (purchased from Wuhan Xavier Biotechnology Co., Ltd.). The RNA concentration was measured using a micro-spectrophotometer (NanoDrop 2000, ThermoFisher, USA), and the A260/A280 ratio was confirmed to be between 1.8 and 2.1 before proceeding. Using the extracted total RNA as a template, reverse transcription was performed following the instructions provided with the reverse transcription kit to synthesize cDNA (G3337-50, Servicebio). A list of primers is provided in Table 2. Real-time polymerase chain reaction (RT-PCR) was performed using CFX Connect RT-PCR Detection System (Bio-Rad, USA). The reaction conditions were as follows: pre-denaturation at 95 ℃ for 30 seconds, 1 cycle; denaturation at 95 ℃ for 15 seconds, annealing/extension at 60 ℃ for 30 seconds, 40 cycles. All reactions were performed using technical triplicates. Expression levels of each gene were recorded, and a 2^-ΔΔCT^ method was employed to calculate gene expression relative to GAPDH. Levene's test for equality of variances was performed, and Welch's t-test (or Student's t-test if equal variances were assumed) was used to calculate statistical significance between cataract and control samples. P-value < 0.05 was considered statistically significant. Bar plots were made using ggplot2.

## Results

### Bioinformatics Analysis

**Figure 2:**
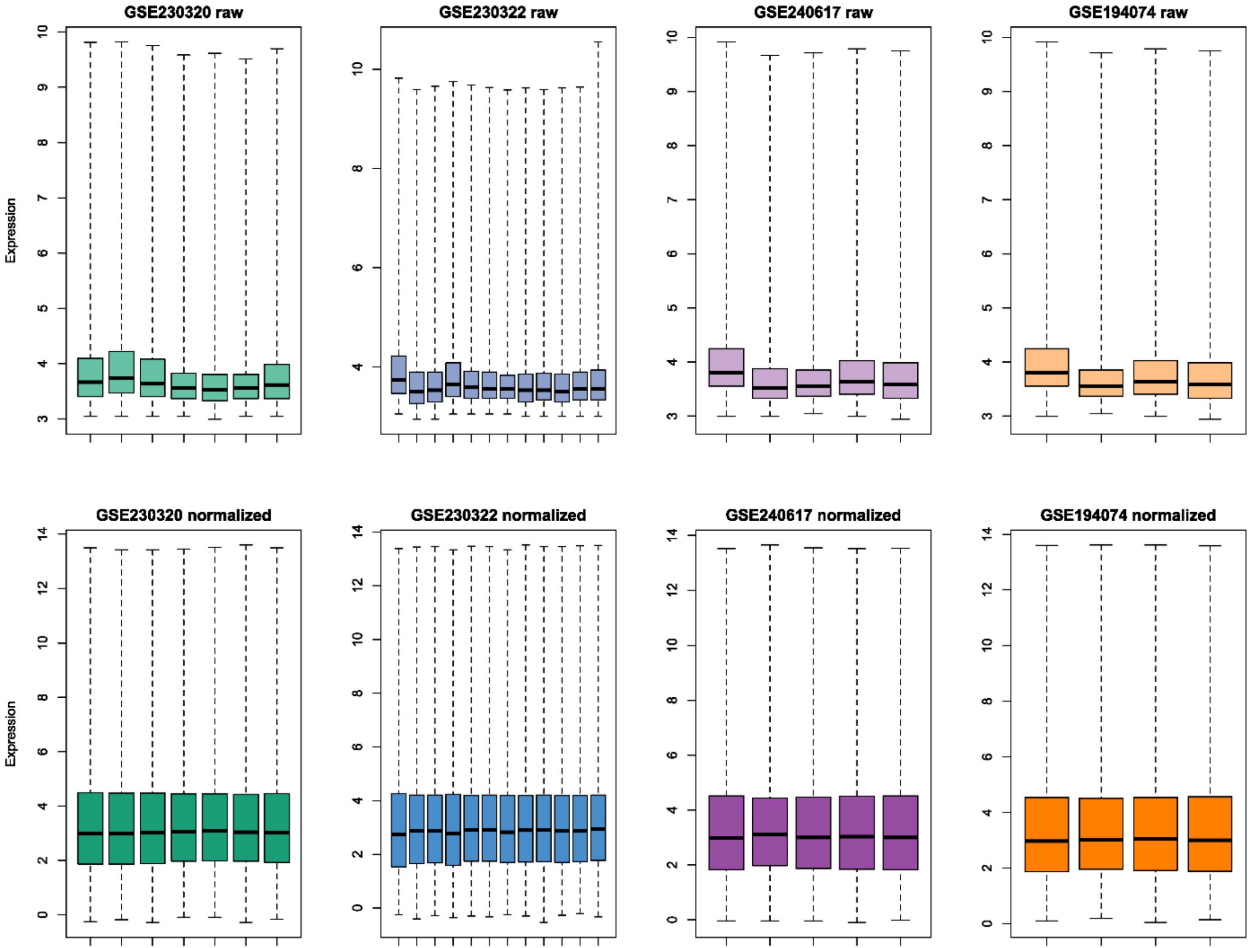
Boxplots of raw and normalized datasets. Whiskers in boxplots extend to extreme points.

**Figure 3:**
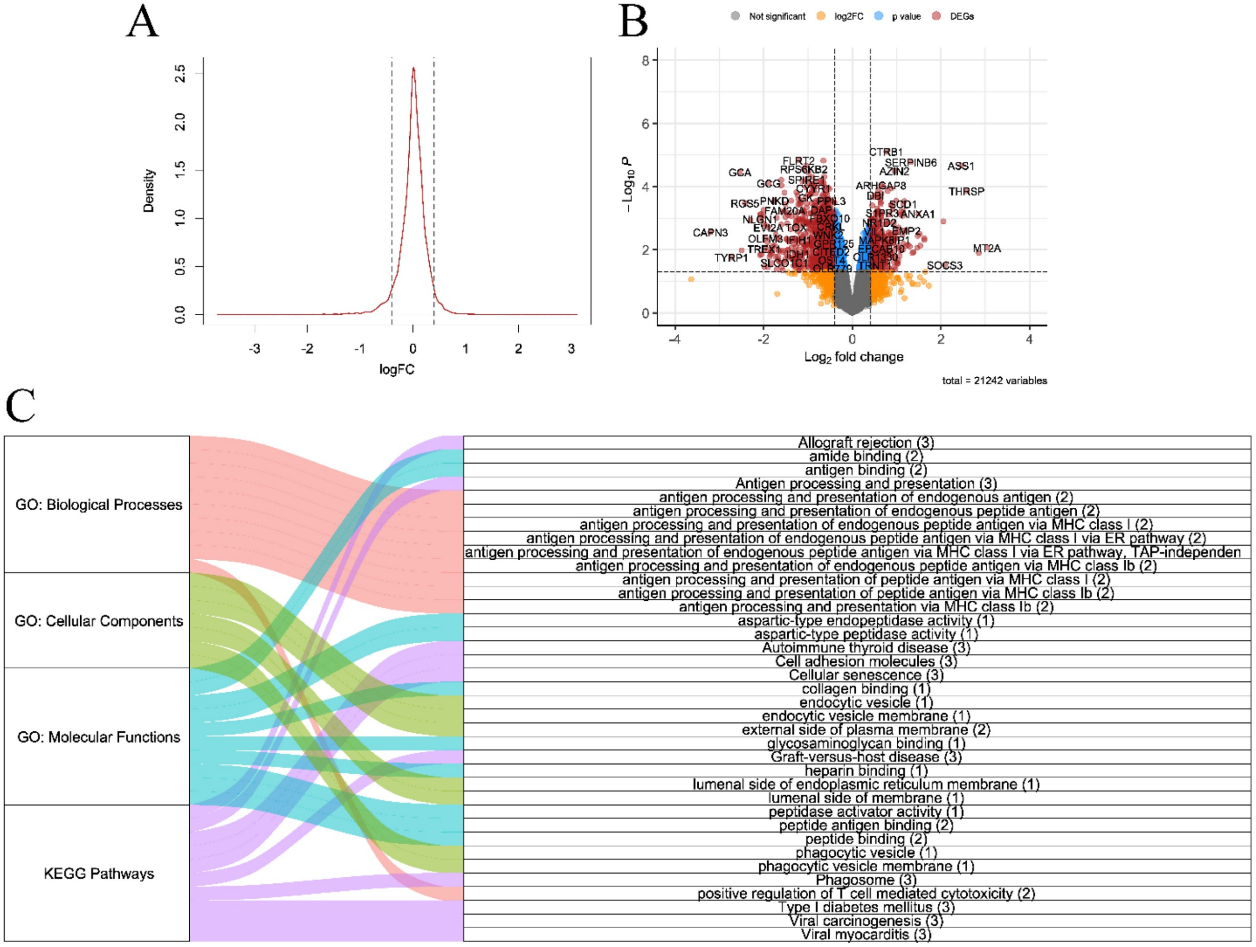
**A.** Density plot of log fold change (FC) of differentially expressed genes **B.** Volcano plot **C.** Alluvial plot of Gene Ontology (biological processes, cellular components, and molecular functions) and Kyoto Encyclopedia of Genes and Genomes enrichment analyses (all P-value < 0.05, gene counts are shown in brackets)

Raw and preprocessed datasets are displayed in Fig. 2. Each dataset initially contained 36685 rows. After all preprocessing steps were complete, 22259 genes remained in each dataset. DEGs screening was performed in the discovery set (GSE230320). A total of 929 downregulated and 438 upregulated DEGs were identified (Fig. 3A-B). According to the results of GO enrichment analysis of biological processes, DEGs were mainly enriched in antigen processing and presentation via major histocompatibility complex (MHC) class I. In addition, genes were predominantly found in endocytic and phagocytic vesicles as well as plasma and endoplasmic reticulum membranes. The identified genes mainly had functions in antigen binding. Based on KEGG enrichment analysis, the genes were enriched in pathways related to graft-versus-host disease, allograft rejection, type I diabetes, and viral carcinogenesis.

### Identification of Relevant Genes

**Figure 4:**
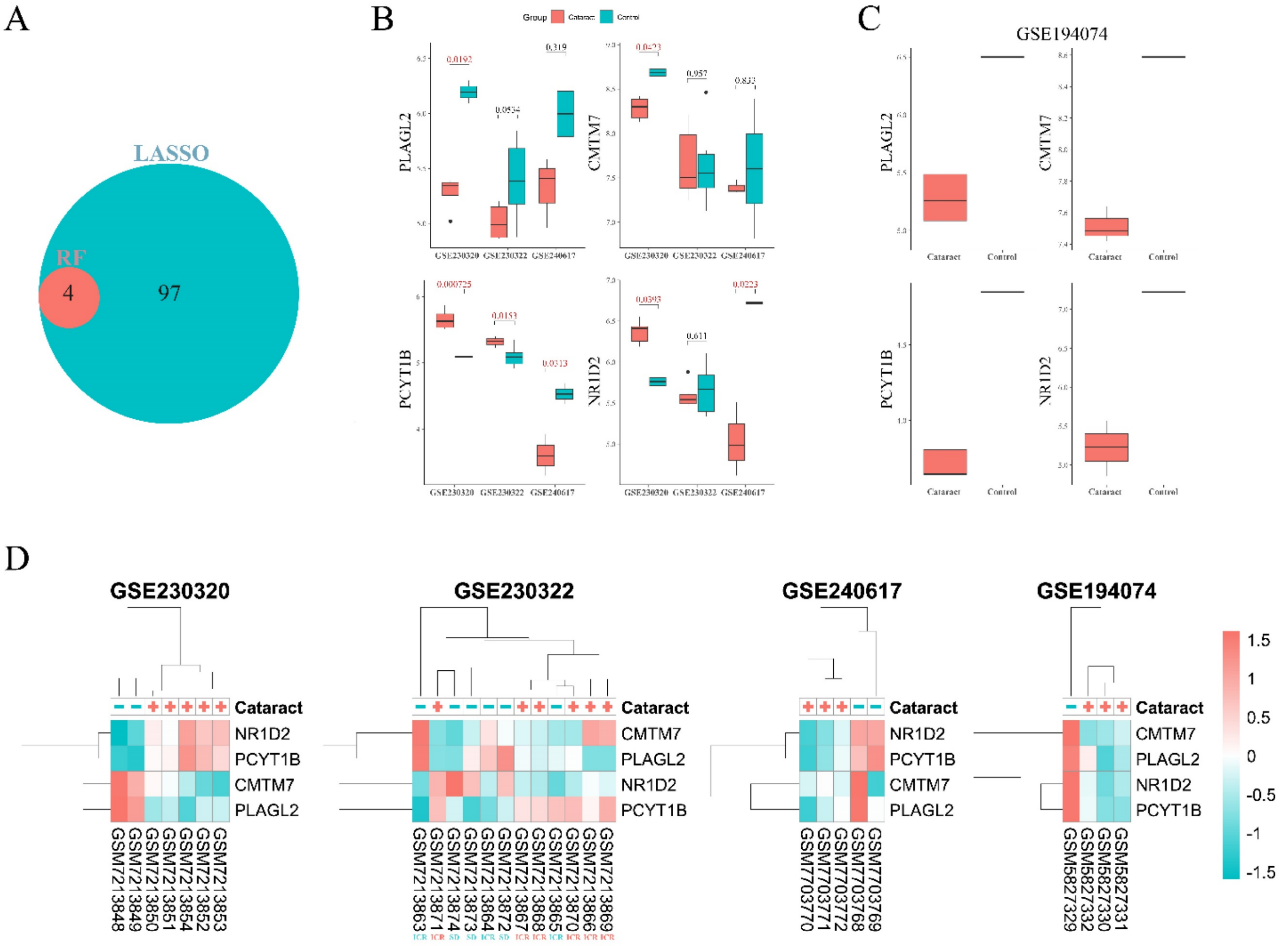
**A.** Intersected genes selected by the Least Absolute Shrinkage and Selection Operator (LASSO) and Random Forest (RF) feature selection tools. **B.** Boxplots of the four overlapped genes (PLAGL2, CMTM7, NR1D2, and PCYT1B) between cataract and non-cataract groups across three datasets (GSE230320, GSE230322, GSE240617). The GSE194074 dataset had only one control sample, and thus t-test could not be performed. Significant differences are highlighted in red. **C.** Boxplots of PLAGL2, CMTM7, NR1D2, and PCYT1B in the cataract and control groups in the GSE194074 dataset. Whiskers: 1^st^/3^rd^ quartile -/+ (1.5*IQR). **D.** Clustered heatmaps of the four genes across all four datasets.

A total of 101 genes were selected using LASSO, and four genes were identified by RF. The intersection of genes selected by these two feature selection tools revealed four overlapping genes: PLAGL2, CMTM7, NR1D2, and PCYT1B (Fig. 4A). The expression levels of these genes were compared between cataract and control groups in the GSE230320, GSE230322, and GSE240617 datasets (Fig. 4B). GSE194074 was excluded from the analysis as it contained only one control sample, making a t-test infeasible. Boxplots of expression levels of the four genes in this dataset are shown in Fig. 4C. Notably, PCYT1B was the only gene that was significantly differentially expressed between the two groups across both *in vivo* and *ex vivo* datasets. Interestingly, in *ex vivo* galactose-induced cataractous lenses, PCYT1B expression was lower compared to controls, whereas the opposite trend was observed in *in vivo* studies. Although NR1D2 was significantly downregulated in the cataract group compared to the control group in the GSE240617 dataset (P = 0.02), its expression did not significantly differ between cataract and control samples in the GSE230322 dataset (P = 0.61). A heatmap was generated to analyze sample clustering and gene expression magnitudes across four datasets (Fig. 4D). The heatmap revealed strong clustering patterns for all datasets except GSE230322, where cataract and control samples were not well separated, with most samples clustering together regardless of their group. For example, a cataractous sample from a 10-week-old ICR rat clustered with a control sample from an SD rat of the same age. Moreover, a control sample from a 4-week-old ICR rat clustered with cataractous lenses from 10-week-old and 8-week-old ICR rats. When comparing expression patterns of CMTM7 and PLAGL2 between the cataract and control groups, similar trends were observed in GSE230320, GSE240617, GSE194074, and selected samples in GSE230322.

### Machine Learning Analysis

**Figure 5:**
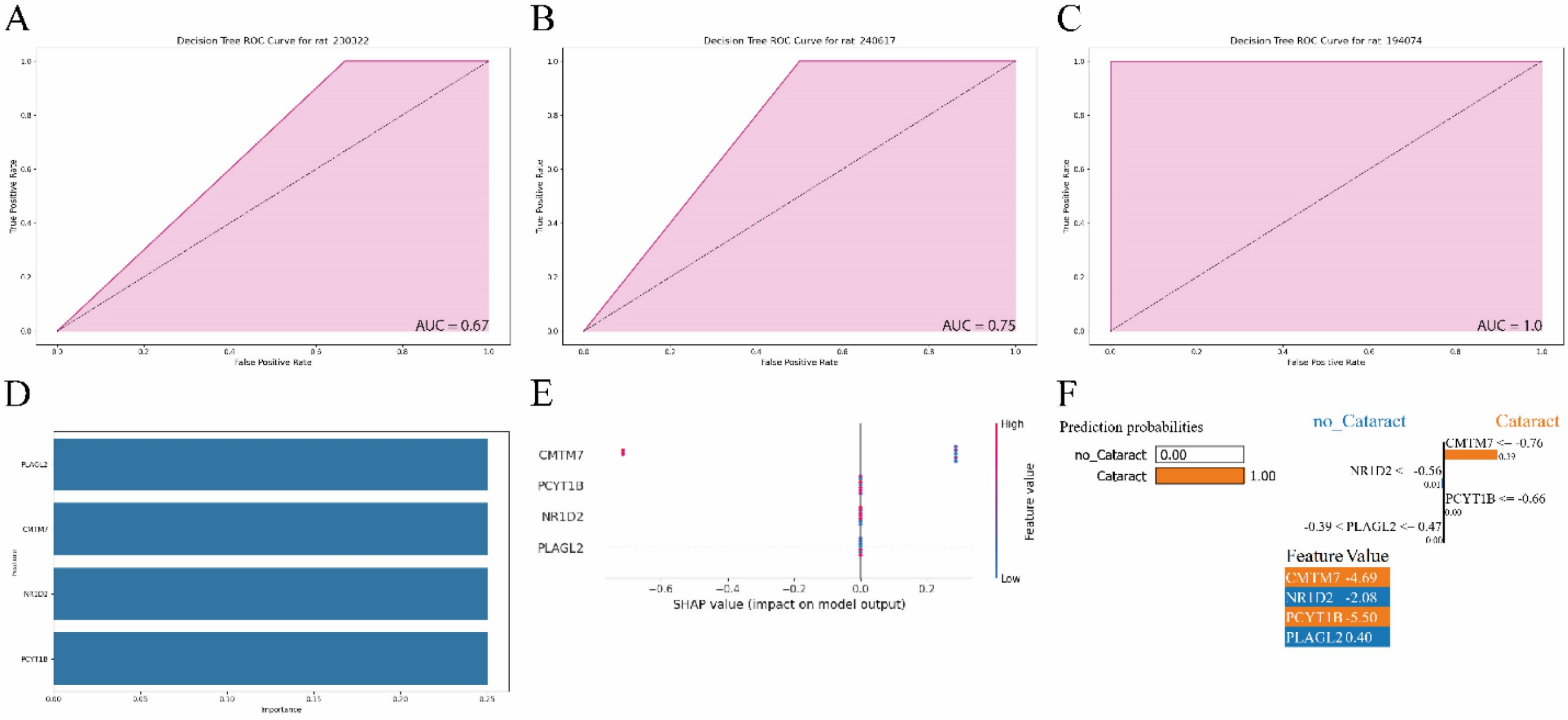
** A-C.** Decision tree receiver operating characteristic (ROC) plots of GSE230322, GSE240617, GSE194074. **D.** Permutation feature importance plot. **E.** SHapley Additive exPlanations (SHAP) plot **F.** Local interpretable model-agnostic explanations (LIME) plot.

DT model was trained on the GSE230320 using PLAGL2, CMTM7, NR1D2, and PCYT1B. The model was validated on one *in vivo* and two *ex vivo* datasets. ROC plots were built to assess the model's performance in each dataset (Fig. 5A-C). All datasets showed good performance, particularly in *ex vivo* datasets with AUC reaching 0.75 and 1.0 in the GSE240617 and GSE194074 datasets.

According to the PFI plot (Fig. 5D), PLAGL2, CMTM7, NR1D2, and PCYT1B had the same feature importance value of 0.25. In our analysis of SHAP values (Fig. 5E), only CMTM7 showed a significant impact on the model's predictions for cataract. Specifically, higher values of CMTM7 were associated with a reduced probability of cataract, as evidenced by two samples with SHAP values of approximately -0.7. Conversely, lower values of CMTM7 in five samples were associated with an increased probability of cataract, with SHAP values around 0.3. The other genes, PLAGL2, NR1D2, and PCYT1B, exhibited SHAP values centered around zero, indicating no substantial contribution to the model's predictions. The LIME figure illustrates that CMTM7 and PCYT1B positively influenced the DT model's prediction of cataracts (Fig. 5F). CMTM7, in particular, had the highest impact, suggesting its critical role in the model's decision-making process. NR1D2 and PLAGL2 had a minor negative influence on predicting “no cataracts”.

### Validation of Gene Expression Levels in Lenses *Ex Vivo*

**Figure 6:**
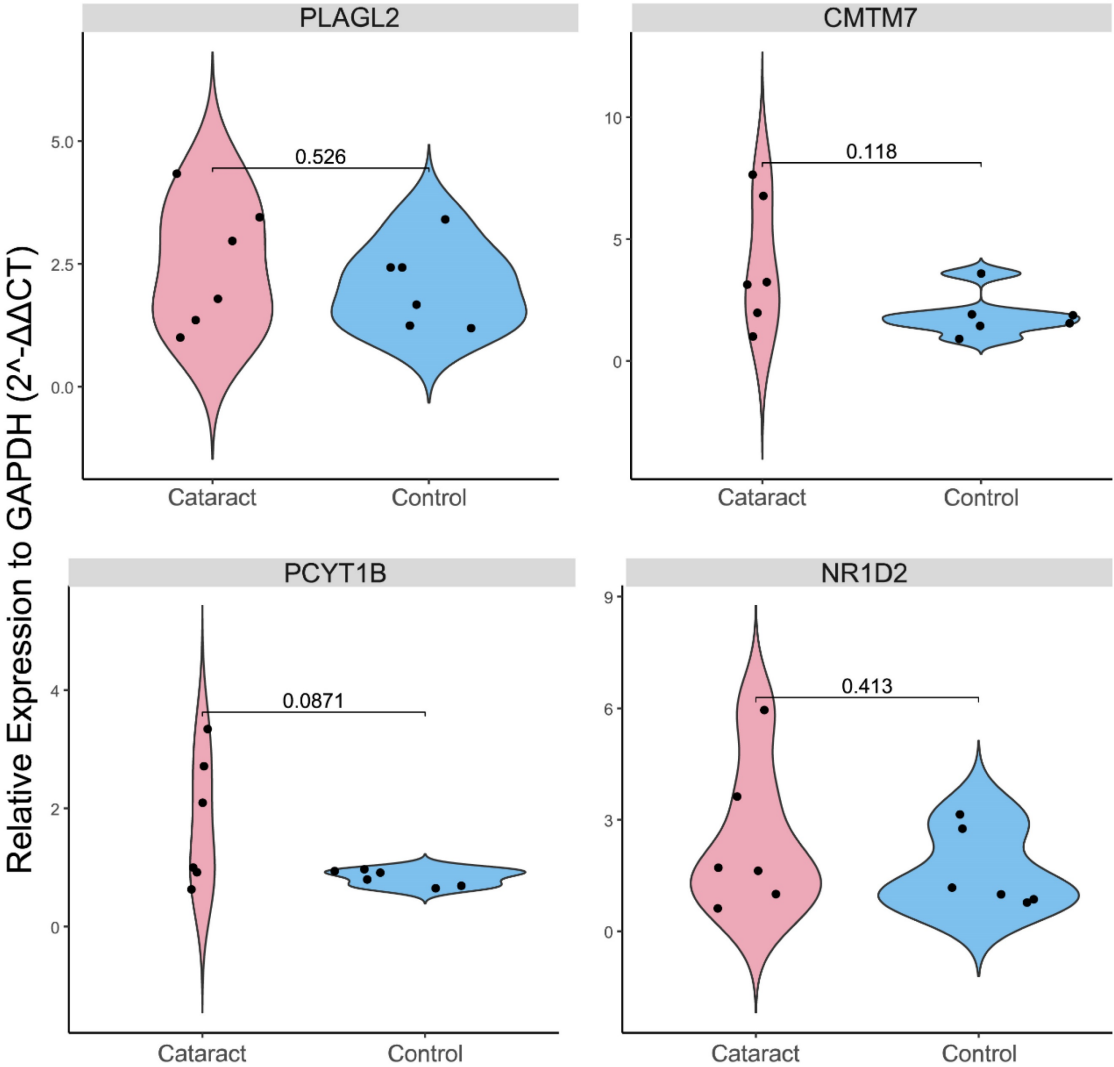
Violin plot of the results of the real-time polymerase chain reaction (RT-PCR) validation of the expression levels (relative to GAPDH based on 2-ΔΔCT method) of PLAGL2, NR1D2, CMTM7, and PCYT1B between control and cataractous (galactose-induced ex vivo) rat lenses.

Expression levels of PLAGL2, CMTM7, NR1D2, and PCYT1B were evaluated in *ex vivo* galactose-induced cataractous rat lenses compared to healthy lenses. After confirming that opacities were successfully induced in all six lenses of the cataract group and no opacities were observed in the six control lenses, an RT-PCR test of each gene was conducted (Supplementary Table 1). Contrary to the earlier findings (Fig. 4B), our results showed no statistically significant differences between the two groups (P-value > 0.05) (Fig. 6).

## Discussion

The identification and interpretation of enriched pathways were challenging due to limited annotations in the rat-specific GO and KEGG databases, as reflected by the low gene counts in each pathway. DEGs were predominantly associated with pathways involved in antigen processing and presentation via the major histocompatibility complex MHC class I, suggesting involvement of immune-mediated processes in the lens tissues of cataract-affected rats^37^. Also, genes were localized to phagosome as well as endocytic and phagocytic vesicles, which implies their presence in these cellular structures rather than indicating an active role in phagocytosis. While phagocytosis is crucial for maintaining retinal cell integrity, particularly in photoreceptor cells^16^, ^38^, it has not been implicated in cataract formation. Thus, the localization of these genes may indicate roles in immune surveillance or other cellular functions related to immune response mechanisms. Moreover, the identified DEGs were enriched in pathways associated with immune responses, including graft-versus-host disease, allograft rejection, etc. Although these conditions are not directly involved in cataract formation in the context of our study, cataractogenesis has been observed as a secondary consequence of retinal allograft rejection in rats^39^-^41^. Therefore, the presence of these pathways in our results likely reflects broader immune activation within the cataractous lenses.

Four genes were selected as the most relevant for DT model training: PLAGL2, CMTM7, NR1D2, and PCYT1B. In brief, the functions of these genes in the eye and their associations with ophthalmic disorders remain unclear. Most research on PLAGL2 and CMTM7 has focused on cancer. PLAGL2 is a potent oncogene^42^-^44^ that promotes cell cycle progression and proliferation, facilitating the transition from the G0/G1 phase to the S phase and subsequent cell division (G2/M)^45^. In contrast, CMTM7 has been reported to exhibit tumor-suppressive effects by affecting the G1/S transition^46^-^48^. Members of the PLAG family, including PLAGL2, are involved in retinal cell differentiation^49^. A paralog of PLAGL2, the PLAG1 gene, has recently been implicated in diabetic retinopathy. It promotes angiogenesis and migration of retinal endothelial cells in a diabetic rat model^50^. NR1D2 is associated with circadian rhythms and lipid metabolism^51^-^54^. Research on NR1D1, a member of the same family as NR1D2, has shown protective effects against retinal inflammation *in vitro*^55^. In another study, activation of NR1D1 resulted in attenuation of retinal pigment epithelial and retinal damage and countered oxidative stress in age-related macular degeneration murine model^56^. Finally, PCYT1B regulates phosphatidylcholine biosynthesis and is predominantly expressed in the brain and reproductive tissues^57^. Knockdown of PCYT1A (a paralog of PCYT1B) in mice has been reported to induce ferroptosis in the retina^58^. Ferroptosis and other forms of cell death play an important role in the progression of various eye disorders, including corneal injury, cataract, glaucoma, etc.^26^, ^58^, ^59^.

A heatmap with hierarchical clustering was created to visualize gene expression patterns across four datasets. Cataract and control groups of all *ex vivo* datasets and the discovery dataset (*in vivo*) clustered rather well. In the GSE230322 dataset, an unusual clustering pattern was observed. Firstly, a sample with cataract from a 10-week-old ICR rat clustered with a sample from an age-matched healthy SD rat. Although SD rats can occasionally develop spontaneous ocular abnormalities, including cataracts, these are rarely severe, especially compared to strains more prone to cataract formation^60^-^62^. Secondly, a control sample from a 4-week-old ICR rat clustered with cataractous lenses from 10-week-old and 8-week-old ICR rats. This unexpected clustering could be attributed to various factors, the most probable of which is the biological characteristics specific to this rat strain. However, the GSE230320 dataset displayed clear and consistent clustering, with no control samples from ICR rats clustering with cataract samples. This suggests that the clustering anomaly in GSE230322 was likely influenced by factors unique to the dataset.

A DT model trained on the *in vivo* dataset (GSE230320), using the genes PLAGL2, CMTM7, NR1D2, and PCYT1B, not only accurately predicted cataracts in the other *in vivo* dataset but also demonstrated good predictive performance in two separate *ex vivo* datasets. Although one gene (PCYT1B) was found to be significantly different between the two groups in the GSE230322 dataset, the overall gene expression patterns between cataract and control samples were very similar in this dataset, as shown in the heatmap. This similarity may be one of the factors that contributed to lower AUC values observed in GSE230322 compared to other datasets. In the *ex vivo* dataset (GSE240617), expression levels of two genes, namely NR1D2 and PCYT1B, were significantly different between cataract and non-cataract groups. Both NR1D2 and PCYT1B displayed opposite expression patterns between the *ex vivo* and *in vivo* datasets. This is likely due to altered environmental conditions, stress responses, and different regulatory mechanisms at play in the two settings. In addition, expression levels of the four genes were validated by conducting RT-PCR of rat lenses. In our laboratory validation, no significant differences in expression levels of all genes were observed between the cataract group and the control group. It is possible that the DT model exhibited good predictive performance across all three datasets, despite the lack of differential expression of most genes in these datasets, due to the model's ability to capture complex interactions between features. Machine learning algorithms, such as DT, do not rely solely on the significance of individual features (in this case, genes) but rather on the combination of features and their interactions^63^, ^64^. Even if the expression levels of individual genes are not significantly different across groups, the model can still identify subtle patterns or interactions between genes that collectively contribute to the prediction of cataract. In other words, the model may have detected small but consistent variations in expression patterns across multiple genes that, when combined, provide a reliable basis for distinguishing cataract from non-cataract samples.

In many settings, DT is a preferred model when it is critical to understand the reasons that lead to a certain prediction^65^. DT model is a particularly effective algorithm for predictive modeling when dealing with gene expression data^66^, ^67^. The algorithm was used to be regarded as unstable and inaccurate^68^, but it has now become one of the most widely used models due to its effectiveness and a relatively low threshold of entry^69^. PFI showed that all four genes had similar importance values in the DT model. However, SHAP and LIME provided different results, identifying CMTM as the most influential gene, with decreased expression correlating with cataract development. The discrepancies between SHAP, LIME, and PFI are due to their differing methodologies. PFI evaluates the impact of a feature by measuring the decrease in model performance when the feature's values are randomly shuffled^35^. This method reflects the overall contribution of a feature to the model's performance but may not capture nuanced interactions or variations across different samples. SHAP and LIME, on the other hand, provide a more nuanced view by considering the effects of features in various contexts and interactions, leading to different interpretations. SHAP values offer a global perspective on feature importance, assessing the impact of each feature across the entire model, which helps in understanding how each gene contributes to the model's predictions on average^70^. LIME focuses on local explanations, providing insights into feature importance for individual predictions. This approach can reveal which features are most influential in specific instances and may highlight interactions and patterns not captured by other methods^71^.

Our study has several limitations. Firstly, similar to other gene expression studies, a major limitation of our research was dataset sparsity. Although we used three external datasets to show the generalizability of our findings, they did not have many samples. The training dataset (GSE230320) had only two control and five cataract samples, whereas two *ex vivo* validation datasets (GSE240617 and GSE194074) had five and four samples, respectively. The second *in vivo* dataset (GSE230322) was larger and contained 12 samples, but it was not used as a training set due to heterogeneous samples. We did not exclude any samples based on rat strain or age to determine whether the model can successfully predict cataracts regardless of age and strain. Secondly, a relatively small number of lenses (six per group) were harvested for PCR validation. This limited sample size could affect statistical power. Thirdly, the RNA purity ratio was between 1.8-2.1. Although a purity of above 1.8 is generally accepted as sufficient for most applications, it could be considered a bit low in this experiment. Fourthly, the same amplification conditions were applied to all genes. Taken together, larger studies are needed to confirm our findings.

In conclusion, although differences in the gene expression of the three out of four selected genes between cataractous and non-cataractous lenses were not statistically significant, the decision tree model trained on the *in vivo* dataset demonstrated strong predictive accuracy for both *ex vivo* and *in vivo* cataracts.

## Supplementary Material

**Supplementary Table 1:** Real-time polymerase chain reaction (RT-PCR) validation of the expression levels of PLAGL2, NR1D2, CMTM7, and PCYT1B in ex vivo rat model.

## Figures and Tables

**Table 1:** Datasets used in this study

Dataset	GSE230320	GSE230322	GSE194074	GSE240617
Model	Galactose-induced cataract			
Experiment type	*in vivo*		*ex vivo*	
Analysis type	Microarray			
Platform	GPL17117			
Details of cataractous lens samples	Five samples from 8- to 18-week-old ICR rats	Six samples from 8- to 10- week-old ICR rats	Three samples from 6-week-old SD rats (incubated for 2-4 days)	Three samples from 6-week-old SD rats (incubated for 2-3 days)
Details of control lens samples	Two samples from 2- and 4-week-old ICR rats	Six samples (three from 4-week-old ICR rats + three from 4-10-week-old SD rats)	One sample from 6-week-old SD rat	Two samples from 6-week-old SD rat
Reference	15		12	17

**Table 2:** List of primers

Gene	Forward primer	Reverse primer	Length (bp)
GAPDH	5'-CTGGAGAAACCTGCCAAGTATG	5'-GGTGGAAGAATGGGAGTTGCT	138
PLAGL2	5'-GTGAAATCTCGGGGACACCAT	5'-GGGTGGCCATGTGCCTATACA	150
NR1D2	5'-TGAGGATGAACAGGAACCGC	5'-GCCAAATCGAACAGCGTCC	86
CMTM7	5'- TGGTAGCCGGAGCGATCTTT	5'-GAGGGGACGGAGAGGCTATG	136
PCYT1B	5'-TGGCCATGCCAGTACTTACC	5'- GCAGTCAGGGTCAGTCGAG	133
