# Supplementary material for: Galactose-Induced Cataracts in Rats: A Machine Learning Analysis
Source: Int J Med Sci. 2025 Feb 10;22(5):1138–49. doi: 10.7150/ijms.103892 (PMC11866541; doi:10.7150/ijms.103892)
Supplement: Supplementary file 1 — Supplementary Table 1: Real-time polymerase chain reaction (RT-PCR) validation of the expression levels of PLAGL2, NR1D2, CMTM7, and PCYT1B in ex vivo rat model. [file ijmsv22p1138s1.pdf]

Supplementary Table 1 Real-time polymerase chain reaction (RT-PCR) validation of the expression levels of PLAGL2, NR1D2, CMTM7, and PCYT1B in *ex vivo* rat model.

			**mRNA expression level**					**Mean value**					**$\triangle CT=CT_{gene}-Ct_{GAPDH}$**				**$\triangle\triangle CT=\triangle CT_{comparison}-\triangle CT_{calibrator}$**				**$2^{-\triangle\triangle CT}$**			
Group	Sample	Experiment	GAPDH	PLAGL2	NR1D2	CMTM7	PCYT1B	GAPDH	PLAGL2	NR1D2	CMTM7	PCYT1B	PLAGL2	NR1D2	CMTM7	PCYT1B	PLAGL2	NR1D2	CMTM7	PCYT1B	PLAGL2	NR1D2	CMTM7	PCYT1B
Cataract	1	1	30.75	36.20	36.87	38.36	35.06																	
Cataract	1	2	31.04	37.16	37.76	37.91	34.40	30.89	37.08	37.42	37.77	34.91	6.20	6.53	6.89	4.02	0.00	0.00	0.00	0.00	1.00	1.00	1.00	1.00
Cataract	1	3	30.88	37.89	37.63	37.05	35.26																	
Cataract	2	1	32.63	37.60	38.23	35.78	34.83																	
Cataract	2	2	32.70	36.97	38.29	36.29	35.53	32.65	37.28	38.49	36.78	35.60	4.63	5.83	4.13	2.95	-1.57	-0.70	-2.76	-1.07	2.97	1.63	6.77	2.09
Cataract	2	3	32.63	37.27	38.93	38.27	36.46																	
Cataract	3	1	32.67	37.17	37.59	38.28	36.61																	
Cataract	3	2	32.66	36.74	36.75	37.28	34.25	32.71	36.79	37.39	37.95	35.29	4.08	4.68	5.24	2.58	-2.12	-1.86	-1.65	-1.44	4.34	3.62	3.13	2.71
Cataract	3	3	32.80	36.46	37.83	38.29	35.01																	
Cataract	4	1	31.02	37.63	38.88	36.90	35.86																	
Cataract	4	2	31.10	36.10	38.22	37.89	35.68	31.10	36.46	38.34	37.01	35.79	5.36	7.24	5.91	4.68	-0.84	0.70	-0.98	0.67	1.79	0.61	1.97	0.63
Cataract	4	3	31.19	35.65	37.92	36.23	35.83																	
Cataract	5	1	32.17	38.27	37.59	38.24	35.41																	
Cataract	5	2	32.60	37.99	38.73	36.96	34.49	32.48	38.24	38.24	37.67	34.76	5.76	5.76	5.19	2.28	-0.44	-0.77	-1.69	-1.74	1.35	1.71	3.23	3.34
Cataract	5	3	32.67	38.45	38.40	37.82	34.38																	
Cataract	6	1	31.78	36.12	35.88	35.59	36.22																	
Cataract	6	2	32.38	36.90	35.92	36.28	36.12	32.05	36.46	36.01	36.00	36.19	4.41	3.96	3.95	4.14	-1.79	-2.57	-2.93	0.12	3.45	5.95	7.64	0.92
Cataract	6	3	31.97	36.35	36.21	36.13	36.22																	
Control	7	1	31.04	34.68	35.28	37.82	35.34																	
Control	7	2	30.84	36.57	36.73	36.90	34.94	30.89	35.31	35.77	37.15	34.95	4.43	4.88	6.26	4.06	-1.77	-1.65	-0.63	0.04	3.41	3.14	1.54	0.97
Control	7	3	30.78	34.69	35.29	36.72	34.56																	
Control	8	1	30.48	36.62	37.51	35.64	35.26																	
Control	8	2	30.74	34.97	36.59	37.61	34.42	30.70	35.62	37.01	36.66	34.85	4.92	6.31	5.95	4.15	-1.28	-0.23	-0.93	0.13	2.43	1.17	1.91	0.91
Control	8	3	30.89	35.27	36.92	36.72	34.88																	
Control	9	1	30.60	35.76	37.62	36.25	35.45																	
Control	9	2	30.55	35.63	37.13	36.80	34.95	30.45	35.91	37.36	36.43	35.00	5.46	6.91	5.98	4.55	-0.74	0.37	-0.91	0.53	1.67	0.77	1.87	0.69
Control	9	3	30.21	36.34	37.32	36.25	34.62																	
Control	10	1	29.22	36.23	37.38	34.62	33.06																	
Control	10	2	29.30	35.14	35.16	34.65	33.75	29.34	35.22	36.09	35.70	33.68	5.88	6.75	6.36	4.35	-0.32	0.22	-0.52	0.33	1.24	0.86	1.44	0.80
Control	10	3	29.48	34.27	35.73	37.82	34.23																	
Control	11	1	30.77	35.24	36.82	36.24	35.33																	
Control	11	2	31.73	36.93	35.29	36.39	36.40	31.06	35.98	36.13	36.11	35.71	4.92	5.07	5.04	4.64	-1.28	-1.46	-1.84	0.62	2.43	2.76	3.59	0.65
Control	11	3	30.69	35.77	36.28	35.69	35.39																	
Control	12	1	29.64	36.19	36.35	37.27	34.02																	
Control	12	2	30.45	35.79	36.83	36.89	34.28	30.05	35.99	36.59	37.08	34.15	5.94	6.54	7.04	4.11	-0.25	0.01	0.15	0.09	1.19	0.99	0.90	0.94
